# Pathogenic Mechanisms of Myeloma Bone Disease and Possible Roles for NRF2

**DOI:** 10.3390/ijms21186723

**Published:** 2020-09-14

**Authors:** Chia-Hung Yen, Chin-Mu Hsu, Samuel Yien Hsiao, Hui-Hua Hsiao

**Affiliations:** 1 Graduate Institute of Natural Products, College of Pharmacy, Kaohsiung Medical University, Kaohsiung 807, Taiwan; chyen@kmu.edu.tw; 2 Center for Cancer Research, Kaohsiung Medical University, Kaohsiung 807, Taiwan; 3 Department of Medical Research, Kaohsiung Medical University Hospital, Kaohsiung 807, Taiwan; 4 Division of Hematology and Oncology, Department of Internal Medicine, Kaohsiung Medical University Hospital, Kaohsiung 807, Taiwan; e12013@gmail.com; 5 Center for Liquid Biopsy, Kaohsiung Medical University, Kaohsiung 807, Taiwan; 6 Department of biology, University of Rutgers-Camden, Camden, NJ 08102, USA; ucdsacnyu@gmail.com; 7 Faculty of Medicine, Kaohsiung Medical University, Kaohsiung 807, Taiwan

**Keywords:** nuclear factor erythroid 2-related factor 2 (NRF2), osteoclast, osteoclastogenesis, osteoblast, multiple myeloma, the receptor activator of NF-kappa B (RANK), the receptor activator of NF-kappa B ligand (RANKL), osteoprotegerin (OPG)

## Abstract

Osteolytic bone lesions are one of the central features of multiple myeloma (MM) and lead to bone pain, fractures, decreased quality of life, and decreased survival. Dysfunction of the osteoclast (OC)/osteoblast (OB) axis plays a key role in the development of myeloma-associated osteolytic lesions. Many signaling pathways and factors are associated with myeloma bone diseases (MBDs), including the RANKL/OPG and NF-κB pathways. NRF2, a master regulator of inflammatory signaling, might play a role in the regulation of bone metabolism via anti-inflammatory signaling and decreased reactive oxygen species (ROS) levels. The loss of NRF2 expression in OCs reduced bone mass via the RANK/RANKL pathway and other downstream signaling pathways that affect osteoclastogenesis. The NRF2 level in OBs could interfere with interleukin (IL)-6 expression, which is associated with bone metabolism and myeloma cells. In addition to direct impact on OCs and OBs, the activity of NRF2 on myeloma cells and mesenchymal stromal cells influences the inflammatory stress/ROS level in these cells, which has an impact on OCs, OBs, and osteocytes. The interaction between these cells and OCs affects the osteoclastogenesis of myeloma bone lesions associated with NRF2. Therefore, we have reviewed the effects of NRF2 on OCs and OBs in MBDs.

## 1. Introduction

Multiple myeloma (MM), characterized by malicious clonal explanation of plasma cells in the bone marrow with creation of monoclonal gammopathy, is the second most prevalent hematologic malignancy in adults. It accounts for approximately 13% of all hematologic malignancies and 1% of all cancers in the world, with a median age of onset of 69 years [1,2]. In addition to plasmacytosis in the marrow and monoclonal protein in serum and/or urine samples, active myeloma is defined by the presence of at least one of the CRAB criteria: Hypercalcemia (calcium >11 mg/dL), renal function insufficiency (creatinine >2 mg/dL), anemia (hemoglobin <10 mg/dL), and bone lesions (1 or more skeletal lesions on survey).

Myeloma bone diseases (MBDs) are characterized by lytic lesions caused by an imbalance of bone remodeling due to an increase in bone resorption and a decrease in bone formation. Myeloma cells interfere with bone remodeling by stimulating osteoclast (OC) function and inhibit osteoblast (OB) differentiation, which results in osteolytic bone lesions of myeloma patient [3,4]. Bone lesions occur in up to 80% of active disease, and complete repair of bone lesions rarely occurs. These lesions are comprised of osteolytic lesions and/or diffuse osteopenia, even in patients without active disease [2,5,6]. In myeloma, bone disease mainly affects the axial skeleton, including the vertebrae (33%), ribs (15%), sternum (13%), and other long bones [7]. It leads to serious complications and/or skeletal-related events (SREs), which include fracture, compression of the spinal cord, hypercalcemia, and the need for further surgical treatment or radiation therapy. SREs not only decrease the quality of life as a result of pain, emotional distress, and treatment procedures, but also affect the survival of MM patients [6,8,9,10]. Therefore, knowledge of myeloma bone lesions for further disease management is warranted.

The interaction among MM cells, OC, and OB relies on many cytokines/proteins from myeloma cells or osteocytes, which promote OC differentiation via the receptor activator of NF-kappa B ligand (RANKL), chemokine C-C motif 3 (CCL3), interleukin (IL)-1,6, and osteoprotegerin (OPG) [11,12,13]. On the other hand, myeloma cells also respond to Wnt signaling pathway inhibitors, like dickkopf-1 (DDK-1) and sclerostin, which inhibit OB function [14]. With the imbalance of bone remodeling due to decreased OB function and increased OC function, MM creates a vicious cycle of tumor expansion and bone destruction.

Current management of MBD such as bisphosphonates (BPs) and denosumab reduce further bone destruction by OCs. Thus, they are therapeutic effects are limited due to the inability to actively stimulate bone formation. Therefore, research into the mechanisms involving in the dysregulation of bone remodeling in MM is essential. Nuclear factor erythroid 2-related factor 2 (NRF2) is a key transcription factor regulates the expression of cytoprotective genes against oxidative and chemical insults. In addition, NRF2 also modulates inflammation responses in several cell types. Recently, NRF2 has been shown to be associated with the malignant phenotype of many cancer cells, including myeloma cells. Interestingly, it also contributes to bone remodeling in disease status. Therefore, we summarized the current knowledge of the pathogenesis of myeloma and myeloma bone lesions. We focused on the inflammatory stress and anti-inflammatory mechanism of NRF2 on bone metabolism in myeloma [15].

## 2. Bone Metabolism

The bone tissue has a distinct composition and function in humans. The primary function of the skeletal system is to provide support and movement of the body and protect the internal organs from damage. It also serves as a mineral reservoir and fat repository. Structurally, bone tissues are composed of a series of canals and cavities interlaced with blood vessels which are all housed in a hydroxyapatite and collagen matrix. This allows a heterogeneous mixture of cells such as bone, immune, mesenchymal, and hematopoietic stem cells to exist in proximity to one another [16]. Bone remodeling is a lifetime dynamic process between bone resorption and formation, which is important for preserving bone integrity, sustaining mechanical loading, and maintaining mineral homeostasis. Bone remodeling arises in a basic multicellular unit, where OCs, OBs, and osteocytes act in a coordinated fashion [17,18]. All bone cells were constituted 90% by osteocytes, while OBs and OCs make up less than 10% of them.

**Figure 1:**
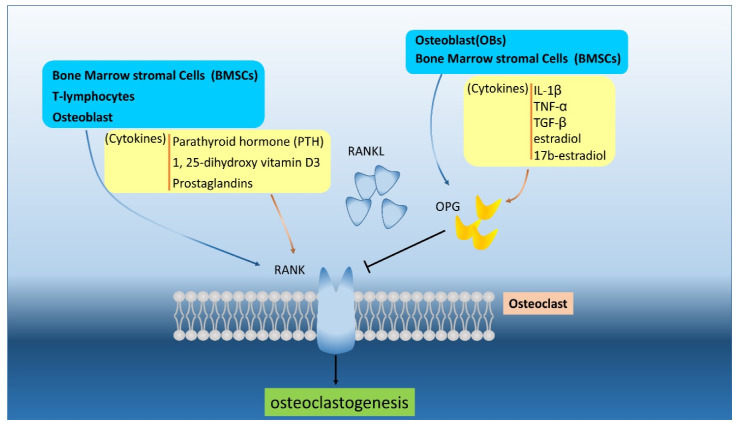
The RANKL/OPG/RANK (receptor activator of NF-kappa B ligand/osteoprotegerin/receptor activator of NF-kappa B) are secreted and regulated by various cells and different cytokines. The RANKL and OPG can be produced by bone marrow stromal cells (BMSCs) and osteoblasts (OBs) to interact or compete RNAK in osteoclast and osteoblast. The OPG can regulated by cytokines, like interleukin (IL)-1β, tumor necrosis factor (TNF)-α, TGF-β, estradiol, and 17b-estradiol.

OCs are multinucleated cells derived from the fusion of mononuclear cells of the monocytes-macrophage lineage originating from hematopoietic stem cells. They contain proteins with bone resorption function, such as tartrate-resistant acid phosphatase (TRAP), carbonic anhydrase II, calcitonin receptors, and some cathepsins (lysosomal protease) [19,20]. In order to decompose the bone matrix, OCs bind to the bone surface strongly enough to create an acidic microenvironment and utilize enzymes like metalloproteinase and cathepsin to degrade said matrix structure. The regulation of this decomposition process depends strongly upon the RANK/RANKL/OPG pathway which is a key regulator for OC differentiation and activity [21,22]. The RANK/RANKL pathway induces downstream signaling pathways for differentiation, development, and maturation [23]. RANK, a transmembrane signal receptor, mainly expressed on the OCs surface and its expression can be induced by cytokines that stimulate bone resorption, such as parathyroid hormone (PTH), 1,25-dihydroxyvitamin D3, and prostaglandins [24]. In a RANK or RANKL knockout mice study, the animal demonstrated severe osteoporosis due to a lack of OCs [25,26]. OPG is a soluble decoy receptor of RANKL, which inhibits osteoclastogenesis through the RANK/RANKL pathway by blockings the interaction of RANK and RANKL [27]. OPG is released by OBs and bone marrow stromal cells (BMSCs) and is regulated by IL-1β, tumor necrosis factor (TNF)-α, transforming growth factor (TGF)-β, and estradiol and 17b-estriol [28,29]. The importance of OPG was demonstrated by the study showing that mice will develop osteopenia and osteoporosis without OPG [13] (Figure 1). The RANKL/OPG ratio is the key for bone osteoclastogenesis; the ratio is low in the normal state, and the ratio becomes abnormal in benign and malignant bone disease [30,31].

**Figure 2:**
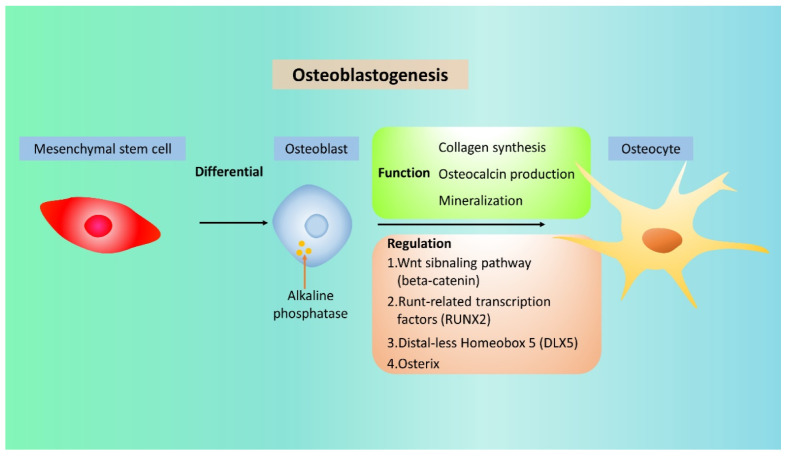
The differentiation and regulation in the osteoblastogenesis process. In the osteoblastogenesis, osteocytes are derived from mesenchymal stem cell then osteoblast which regulated by beta-catenin, Runt-related transcription factor 2 (RUNX2), Distal-Less Homeobox 5 (DLX5), and osterix. The collagen synthesis, osteocalcin production, and mineralization let the osteoblasts formulate the osteocytes.

OBs are mononuclear cells originating from mesenchymal stem cells and evolve into osteocytes or bone-lining cells by specific steps [32]. They contain the enzyme alkaline phosphatase for bone formation, which can also be used as a marker of OB activity [33]. They are normally located on the bone surface and lay down new bone [34]. Through collagen formation and the production of osteopontin and osteocalcin, they mineralize the bone matrix and solidifies the bone. The specific differentiation steps of OBs are modulated by the time-dependent expression of transcription factors, such as Runt-related transcription factor 2 (RUNX2), Distal-Less Homeobox 5 (DLX5), and osterix, in the osteoblastogenesis process [32] (Figure 2). Osteoblastogenesis relies on the balance of agonist and antagonist activity of the Wnt signaling pathway, which regulates the expression of the transcription factor beta-catenin [35]. After new bone formation, OBs may serve as lining cells on the bone surface, become osteocytes, embed itself into the mineralized matrix, or simply undergo apoptosis [36].

Bone remodeling is a continuous balance between bone absorption and bone formation [37]. Osteocytes serve as the main regulators of bone homeostasis between OCs and OBs [38]. In the bone, osteocytes detect microcracks, mechanical strains, and alternations in the hormonal milieu, and respond these changes by bone remodeling [39,40]. They control the activity of OCs and OBs by secreting the regulators of these cells, such as RANKL, OPG, and sclerostin [41,42]. Through these responses, osteocytes play a key role in bone homeostasis.

## 3. Myeloma Induced Bone Disease

In myeloma patients, the disrupted OB-OC-osteocyte axis leads to unbalanced bone remodeling with a dramatic change in osteolytic bone lesions [43,44]. Dysregulation of OCs and OBs is the key mechanism of myeloma bone lesions, in particular, the upregulation of osteoclastogenesis, inhibition of OB activity, and increase in apoptosis of osteocytes [36,45,46]. In turn, the growth factors released by the increased bone resorption process regulate the survival, proliferation, and drug resistance of myeloma cells, creating a vicious cycle of myeloma expansion and myeloma bone lesions [46,47]. Since the pathogenesis of MBDs is primarily caused by generalized activated OC activity, there is a relationship among tumor burden, disease activity, OC numbers, and resorption status in myeloma patients.

There are many signaling factors in MM patients that promote OC differentiation and activity, such as RANKL, chemokines (CCL3), IL-6, and myeloma-derived exosomes [48,49]. These signaling factors promote and stimulate the migration, survival, and differentiation of OC precursor cells [50,51]. Additionally, myeloma cells modify the surrounding microenvironment and inhibit osteogenesis by direct secretion of Wnt antagonists, such as DKK-1, and by inducing the OB inhibitors, sclerostin, and activin, from mesenchymal cells and osteocytes [35,37]. Osteocytes play a role in myeloma bone lesions as the number of osteocytes is reduced in myeloma patients [52,53] as a result of apoptosis in myeloma disease. The apoptotic osteocytes express high levels of RANKL and sclerostin, which further inhibits OB differentiation and attracts OC precursors. The adherence of myeloma cells to BMSCs by binding to vascular cell adhesion molecule-1 (VCAM-1) also stimulates the release of many factors that regulate osteoclastogenesis and osteoblastogenesis [14,46]. These factors include macrophage-colony stimulating factor (M-GCSF), IL-1, IL3, IL6, IL17, and macrophage inflammatory protein 1 (MIP-1-alpha/CCL3), TNF-alpha and beta, parathyroid hormone-related protein (PTHrP), and vascular endothelial growth factor (VEGF). The myeloma and the microenvironment orchestrate these factors and increase the RANKL/OPG ratio in the development of osteolytic bone lesions in myeloma [30].

## 4. The RANK/RANKL Pathway

**Figure 3:**
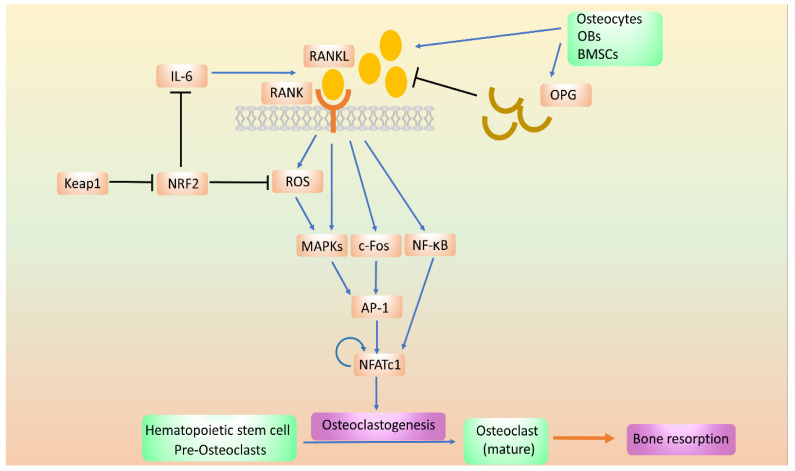
The regulation of signal pathways in osteoclastogenesis. In osteoclast (OC) differentiation, the RANKL/RANK pathway that regulate the downstream intracellular markers such as mitogen-activated protein kinases (MAPKs), c-Fos, and NF-κB, which contribute the OC maturation. In the pathway, NRF2 could reduce the osteoclastogenesis by ROS inhibition.

The RANK/RANKL pathway is the most relevant physiological and therapeutic signaling pathway for the bone resorption regulation [51]. RANK, a subgroup of the tumor necrosis factor (TNF) family, is a transmembrane signal receptor that is mainly expressed on the surface of OCs [53]. RANK ligand (RANKL) is the associated cytokine in the microenvironment that interacts with RANK for bone resorption and is mainly secreted by osteocytes, and to a lesser extent, by BMSCs and OBs. Through the binding of RANKL to RANK on immature OCs, RANKL induces the differentiation of OCs into mature OCs, which are the main regulator of osteoclastogenesis [54]. Upon the binding of RANKL to its receptor RANK, several signaling pathways for osteoclastogenesis are triggered, including the expression of nuclear-factor-activated T-cell cytoplasmic 1 (NFATc1) [55,56,57]. NFATc1 is the master regulator of OC differentiation that particulates in many pathways; the activation also triggers NF-κB and mitogen-activated protein kinase (MAPK) and subsequent pathways, including INK, p38, ERK, and Src [57,58]. It promotes the expression of OC-specific genes, including tarftrate-resistant acid phosphatase (TRAP), OC stimulatory transmembrane protein (OC-STAMP), cathepisn K, and MMP9, which induce bone resorption. RANKL also triggers the expression of c-Fos, a major component of the activator protein-1 transcription factor complex, which induces the expression of NFATc1 [59,60] (Figure 3).

In contrast to RANKL, osteoprotegerin (OPG), a decoy receptor of the TNF family, is secreted by OBs and acts as a RANK antagonist by binding to RANKL, which inhibits osteoclastogenesis [61]. An increase in the RANKL/OPG ratio was noted in both inflammatory diseases and several cancers, which resulted in the loss of bone mass [62]. An imbalance in the ratio was also found in myeloma patients with increased RANKL expression and a decrease in OPG expression in the marrow environment, which shows that the ratio of RANKL/OPG plays a critical role in bone metabolism in myeloma.

In myeloma status, the plasma cell increases RANKL expression by direct secretion and by myeloma stromal cells through direct cell-to-cell contact and by osteocytes [63]. On the other hand, myeloma cells decrease OPG levels by reducing the secretion of OPG from OBs and stromal cells. In addition, upregulation of cytokines, such as IL-1, IL6, TNF-α, and hormones, such as PTHrp, increases RANKL expression and decreases OPG levels through the contact of plasma cells and stromal cells, and the myeloma microenvironment [64]. In particular, myeloma cells induce osteocyte apoptosis by activation of the NOTCH pathway, and the apoptosis process of osteocytes induces high levels of RANKL [65,66].

## 5. Introduction of NRF2 (or Structure, Function, and Regulation of NRF2)

Nuclear factor erythroid 2-related factor 2 (NRF2) is a transcription factor in numerous cell types [67]. It belongs to a basic leucine zipper protein family (bZIP), which is comprised of 4 members, including NRF1, NRF2, NRF3, and P45 NF-E2 [68]. In these bZIP transcription factors, NRF1 and NRF2 are ubiquitously distributed, the expression of NRF3 is restricted to the liver and placenta, and the expression of NF-E2 is limited to erythrocytes [69]. NFR2 is considered as the main mediator of cellular adaptation to redox stress among these members [70,71]. From its protein conformation of N- to C-terminus, NRF2 has seven functional domains, arranged as NRF2-ECH homology (Neh) domain 2 (Neh2), Neh4, Neh5, Neh7, Neh6, Neh1, and Neh3 [72,73,74]. Neh2 domain contains specific sequences for binding with the negative regulators Keap1 that mediate the degradation of NRF2. The Neh4 and Neh5 form the transactivation domain of NRF2 that recruits CREB-binding protein (CBP) and receptor-associated coactivator 3 (RAC 3). Neh7 domain interacts with retinoid X receptor alpha (RXRα), which represses the transactivation activity of NRF2. Neh6 domain contains two conserved redox-independent degrons known as DSGIS and DSAPGS motifs, which can be recognized and phosphorylated by glycogen synthase kinase-3β (GSK-3β), then targeted for degradation by β-transducin repeat-containing protein (β-TrCP) E3 ubiquitin ligase. Neh1 is a CNC (cap ‘n’ collar)-basic leucine zipper domain that mediates binding to DNA and dimerizing with small musculoaponeurotic fibrosarcoma (sMaf) proteins. The C-terminal Neh3 domain interacts with the chromo-ATPase/helicase DNA-binding protein 6 (CHD6) is important for the transcriptional activity of NRF2.

NRF2 is a master regulator to maintain cellular redox homeostasis and regulates the expression of a large cytoprotective network, which include enzymes involved in the biosynthesis, utilization, and regeneration of nicotinamide adenine dinucleotide phosphate (NADPH), thioredoxin, and reduced glutathione (GSH). In addition, detoxification enzymes, such as glutathione S-transferase (GST), aldo-keto reductases, and NAD(P)H: Quinone osidoreductase 1 (NQO1) as well as the proteins participate the recognition and clearance of damaged proteins and organelles, such as proteasomal subunits and autophage-related proteins [70,75,76,77]. The main functions of the products of NRF2-related genes are to neutralize electrophiles and reactive oxygen/nitrogen species (ROS/RNS), thus reduce intracellular oxidative stress that protect the cell from harmful insults of these agents [77,78]. NRF2 is also involved in the regulation of a number of metabolic genes, such as the biosynthesis of purine nucleotides, the pentose phosphate pathway, and enhancement of mitochondrial function and fatty acid metabolism [79].

As one of the master regulator factors of cytoprotective functions, NRF2 is regulated mainly by the level of protein stability. At homeostasis, the level of NRF2 is low as a result of continuous ubiquitination and proteasomal degradation. However, under stress the NRF2 is able to accumulate eventually allowing it to bind with sMaf in the nucleus and initiate the transcription of its target.

KEAP1, a cytoplasmic actin-binding protein, tethers NRF2 in the cytoplasm and represses the activity of NRF2. In addition to being a repressor of NRF2 activation, KEAP1 serves as the intracellular sensor of electrophiles and oxidants, which induce stabilization and activation of NRF2. When exposed to either electrophiles or oxidants, KEAP1 is modified on its cysteine residues, leading to the release of NRF2 from KEAP1. This action subsequently leads to the translocation of NRF2 in the nucleus. Upon translocation into the nucleus, activated NRF2 forms heterodimers with sMaf proteins, and dimerization is necessary for DNA binding [80,81]. The NRF2-sMaf then binds to the regulatory area of antioxidant response elements (ARE), a cis-acting enhancer sequence located in the promoter regions of genes encoding for antioxidants and detoxifying enzymes [82], such as GSTs, NADPH-quinone oxidoreductase (NQO) 1, and glutamate-cysteine ligase catalytic (GCLC) subunit [83,84]. ARE comprises the core sequence 5′-TGACnnnGC-3′, which is critical for binding.

There are three ubiquitin ligase systems that are implicated in the NRF2 degradation. KEAP1 is the main negative regulator of NRF2 activity. Under homeostasis, NRF2 interacts with its cytosolic repressor, KEAP1, a subunit of the Cullin 3-RING Box 1 (CUL3-RBX1) E3 ubiquitin ligase complex that primes NRF2 for proximal degradation [85,86]. The second ubiquitin ligase system is mediated by β-TrCP-Cullin 1-RING Box 1 (CUL1-RBX1) E3 ubiquitin ligase complex in a KEAP1-independent manner. Nuclear NRF2 can be targeted and phosphorylated by GSK-3β in Neh6 domain, which creates a degradation domain that is then recognized by β-TrCP and tagged for proteasome degradation by a CUL1-RBX1 E3 complex [87,88]. In line with the finding, genetical or pharmacological inhibition of GSK-3β results in an increase in NRF2 protein levels. GSK-3β inhibition-mediated NRF2 stabilization occurred in embryonic fibroblast from KEAP-knockout mice and in cell ectopically expressed NRF2ΔETGE mutant which cannot bind to KEAP1, further demonstrated that GSK-3β degrades NRF2 in a KEAP1-independent manner [88,89]. The third pathway controlling NRF2 stability has been reported in cirrhotic levers with the protein HRD1, an E3 ubiquitin ligase associated with the ER membranes. HRD1 is transcriptionally induced by ER stress; HRD then interacts with the Neh4-5 domains of NRF2 and promote its ubiquitination and degradation [73].

Autophagy is generally believed to degrade long-lasting, misfolded proteins, and damaged organelles via lysosomal pathway. Autophagy was found to be directly linked to NRF2 through an adaptor protein sequestosome 1 (SQSTM1)/p62 [90]. A KEAP1 interacting region (KIR) in p62 binds to KEAP1 in the same basic domain as NRF2, therefore competes with NRF2 for binding to KEAP1 and leads to NRF2 accumulation and subsequent transcriptional activation of its target genes [90]. In addition, upon binding to KEAP1, p62 can induce autophagic degradation of KEAP1. Interestingly, p62 can be induced under oxidative stress condition in an NRF2-dependent manner, thus creating a positive feedback loop between NRF2 and p62 [91].

## 6. The Role of NRF2 in the Myeloma Microenvironment

MM is the result of neoplastic transformation from a terminally differentiated B lymphocyte. As such, it primarily exists and proliferates in the bone marrow similar to other normal plasma cells [92,93,94]. The interaction of plasma cells with the surrounding bone marrow environment functionally and physically is important for the proliferation and survival of plasma cells [95]. Two compartments in the bone marrow microenvironment interact with plasma cells, which promote proliferation, survival, and even drug-resistance of myeloma cells [92,93,94]. One is the cellular part comprising hematopoietic cells and non-hematopoietic cells, such as fibroblasts, OBs, mesenchymal stem cells, myeloid-derived suppressor cells (MDSCs), and BMSCs. Soluble factors, such as cytokines, chemokines, growth factors, and extracellular matrix proteins, comprise the non-cellular part that also interact with myeloma cells.

For the soluble factors, IL-6 is one of the most critical factors for myeloma cell survival, proliferation, and drug resistance [96,97]. In the marrow milieu, many cells including MDSCs, fibroblasts, macrophages, adipocytes, and dendritic cells, secrete IL-6. IL-6 has been shown to increase RANKL protein expression in murine osteoblasts and BMSCs, and been considered as a bone resorption cytokine [98]. Elevated IL-6 can be attributed to the activation of NF-κB signaling in many cell types [99]. Suppression of NRF2 and activation of NF-κB were observed in pathological conditions, such as inflammation [100]. NRF2 competes with NF-κB for downstream signaling, such as with co-activator CBP/p300, and the activated NRF2-suppressed NF-κB signaling [101]. Therefore, the expression of NRF2 is associated with a decrease in IL-6 expression in several cell types [102]. In NRF2-deficient mice, macrophages and embryonic fibroblasts showed higher expression of NF-κB and production of inflammatory cytokines IL-6 [103,104].

Additionally, vascular cell adhesion molecule-1 (VCAM-1) is also a target of NF-κB. As such, the suppression of NF-κB signaling in BMSCs results in decreased expression of ICAM-1 and VCAM-1, which affects the resistance of MM to proteasome inhibitors [105,106]. Likewise, activation of NRF2 could suppress the adhesion molecules expression in BMSCs by acting as an antagonist of the NF-κB pathway [106]. Activation of NRF2 by curcumin and dimethyl fumarate inhibits the expression of NF-κB and adhesion molecules in animal models [107]. These findings suggested that NRF2, through inhibiting the transcription activity of NF-κB, could suppress both the soluble factor (e.g., IL-6)-mediated drug resistance and cell-adhesion-mediated drug resistance. [15,108]. Given the critical role of IL-6 plays in RANKL expression, the effect of NRF2 on RANKL expression/secretion in the bone marrow microenvironment is worth pursuing.

## 7. The Role of NRF2 in Bone Metabolism

Since oxidative stress affects bone homeostasis, it is reasonable to suppose that the NRF2 signaling pathway plays a role in bone metabolism [109,110]. ROS originates from aging, hormone imbalance, and insults from radiation and trauma as well as cancers has adverse effects on bone structure and lead to bone fragility [14,46]. In contrast, antioxidant defenses significantly decrease the osteoporotic status of postmenopausal women. This stresses the important role of the balance between oxidants and antioxidants in the maintenance of bone metabolism between OCs and OBs. As a master regulator of antioxidant response system in the body, and defect of NRF2 leads to a deficiency in the production of numerous antioxidant enzymes and glutathione and an increase in intracellular ROS levels in both OC and OB precursor cells [74,109,111]. The increase in osteoclastogenesis and decrease in osteoblastogenesis has been noted to be associated with an imbalance between the plasma antioxidant and oxidant biomarkers in postmenopausal osteoporotic women [111]. These findings highlight the role of oxidative stress in bone metabolism.

In animal studies, deletion of NRF2 in bone leads to a lower mineral density, and the bone strength of vertebral and femurs bodies was significantly lower than that in normal control group [112]. The lower bone mass and bone strength in NRF2-deficient mice were resulted from the imbalance of bone formation and resorption [113] specifically in relation to bone remodeling, which includes co-ordination of OC and OB cells continuously and/or sequentially [114]. The remodeling of bone caused by the imbalance of OC and OB activity induces the removal and restoration of bone tissues [109]. These studies suggest NRF2 partakes in a critical role in bone metabolism.

## 8. The Effects of NRF2 on OC

OCs are multinucleated cells derived from hematopoietic stem cells. Accumulation of osteoclastogenesis results in bone destruction of bone lesions in inflammatory disease and malignant status [115,116]. RANK/RANKL is the key inducer of OC differentiation and control of osteoclastogenesis [54]. Produced by OB and fibroblast cells, RANKL is expressed in the destructive stage of rheumatoid arthritis with an increase in osteoclastogenesis [116]. In cell line studies, expression of RANKL decreased the ratio of NRF2/KEAP1, which decreased the expression of NRF2-related enzymes and favored the increase in ROS activity [117,118]. ROS is a downstream molecular signal of RANKL and is associated with OC differentiation [119]. Since a reduction of ROS inhibits OC activity and osteoclastogenesis, a depletion of NRF2 will result in an elevated level of intracellular ROS from the removal of the antioxidant suppressing ability of NRF2 therefore leading to an increase in the quantity of OCs and osteoclastogenesis [120]. Moreover, overexpression of NRF2 directly or by inhibition of KEAP1 decreases the expression of RANKL, which agreed with the notion that NRF2-deficient induced osteoclastogenesis could result from elevated RANKL expression in bone marrow microenvironment [112,119,121]. In addition, NRF2 could influence osteoclastogenesis by the expression of IL-6 [122]. These data suggested that NRF2 is crucial to OC activity, but the underlying mechanism of NRF2 in regulating OC activity remains unclear [123]. Increased p38 MAP kinase activity but not the NF-kB pathway was associated to ROS elevation in NFR2 knockout mice [124,125,126,127]. Moreover, NRF2 also represses OC activity via regulating the activity of NFATc1, which is the master transcription factor for osteoclastogenesis [124,128]. Despite of these findings suggesting a suppressive role of NRF2 in osteoclastogenesis and OC activity, it is still not clear whether the same effects of NRF2 can be observed in MBD. It has been shown that the levels of oxidative stress biomarkers were significantly higher in MM patients with bone lesions compared with those without lytic bone lesions [129]. Moreover, RANKL is the major regulator of pathological bone remodeling in MBD. Thus, it is important to compare the ROS level and NRF2 activity in OCs in MM patients with and without bone lesions, and in MBD mouse model.

## 9. The Role of NRF2 in OB

The relationship of NRF2 in the activity and differentiation of OBs remains controversial [109]. The NRF2 knockout mice study reported by Sun and colleagues showed lower bone mass not only from increased bone resorption but also from reduced bone formation with a lower mineral apposition rate [112]. These data support that NRF2-deficient OBs lose their ability to differentiate and mineralize, which decreases bone formation with reduced bone mass.

The role of NRF2 in regards to OBs may also be related to the level of intracellular ROS, as a higher level of ROS has been observed in NRF2 deficient OBs and stromal cells. This increased level of oxidative stress from elevated ROS levels results in the inhibition of OB differentiation [121]. NRF2 levels in OBs also interact with osteoclastogenesis because a decrease in ROS levels by NRF2 activation leads to a reduction in RANKL, which inhibits OC activity and osteoclastogenesis [121]. NRF2 activation also reduced IL-6 secretion, leading to suppression of osteoclastogenesis [122]. Therefore, it is presumed that the activated NRF2 could promote osteoblastogenesis and suppress osteoclastogenesis.

However, overexpression of NRF2 caused deleterious effects on OBs since overexpression of NRF2 inhibits RUNX2 in OB cells [130,131]. This inhibition of RUNX2 presents a problem as it is a master transcription factor that regularize embryonic bone development and postnatal OB functions [132,133]. RUNX2 deficiency causes suppression of endochondral and intramembranous bone formation and arrest of chondrocyte maturation [134]. Although, RUNX2 levels should be kept in an ideal range since both RUNX2-deficient mice and animals with RUNX2 overexpression presented with osteopenia [135,136]. This implies that many transcription factors in bone acquired a narrow expression range for physical functions [135]. These data suggest that the role of NRF2 in OBs, such as those in regards to RUNX2, is complicated and depends on many factors, such as age, sex, and physiological versus pathological conditions, and an optimal range for bone formation [109,135]. Which further demonstrates the important role that NRF2 has in the mechanism for bone formation.

## 10. The Role of NRF2 in Mitochondrial Regulation of Bone Homeostasis

In addition to the regulation of proteins involved in oxidative response and xenobiotic detoxification and the inhibition of inflammation, recent evidence indicated that NRF2 also affects mitochondrial functions [137,138]. As a center where multiple redox reactions take place, mitochondria are the principal source of ROS. By transcriptional regulating of several antioxidant proteins, NRF2 maintains the redox homeostasis of mitochondria. Antioxidant enzymes involving in GSH biosynthesis including γ-glutamyl cysteine ligase catalytic (GCLC) and modulatory (GCLM) subunits as well as glutathione reductase (GR) are induced by NRF2 and participates in the maintenance of the mitochondrial GSH pool [139]. NRF2 also activates the expression of glucose-6-phosphate dehydrogenase (G6PD) and 6-phosphogluconate dehydrogenase (6PGD) which are key enzymes in the pentosephosphate pathway (PPP). One important function of PPP is producing NADPH, a cofactor that is required for the generation of reduced GSH [140]. Furthermore, mitochondrial antioxidant enzymes such as superoxide dismutase 2 (SOD2), GSH peroxidases 1 (GPx1), thioredoxin reductase 2 (TRXR2), peroxiredoxin 3 (PRDX3), and PRDX5 have been shown to be upregulated in NRF2 dependent manner [138].

NRF2 also participates in mitochondrial biogenesis and oxidative phosphorylation (OXPHOS). Which along with Nuclear respiratory factor-1 (NRF-1) and mitochondrial transcription factor A (TFAM) are central factors in controlling mitochondrial biogenesis [141]. Piantadosi et al. demonstrated that in cardiomyocytes activates NRF2 upregulates NRF-1 and subsequently activates mitochondrial biogenesis genes [142]. Additionally, evidence provided by Wu et al. indicated activated NRF2 binds to the promoter of TFAM and enhances mitochondrial biogenesis [143]. In addition to mitochondrial biogenesis, NRF2 has also been shown to modulate mitochondrial function directly [144]. Holmström et al. showed that cells and mitochondria isolated from NRF2-knockout mice displayed lower levels of respiration and ATP production. Conversely, those processes were increased in mice counterparts with genetically activated NRF2 (KEAP1-knockout or KEAP1-knockdown) [145]. In addition, the reduction of respiration and ATP levels in NRF2 deficiency condition could be a result of the limitation of substrate. Fatty acid oxidation (FAO) produces acetyl-CoA which fuels the TCA cycle, and then enhances the generation of FADH_2_, and NADH, which are major electron donors in OXPHOS. Interestingly, FAO capacity was reduced in cells and mitochondria isolated from NRF2-knockout mice [146]. Moreover, NRF2 depletion diminished the expression of carnitine palmitoyltransferase (CPT), a rate-limiting enzyme in FAO, while pharmacological activation of NRF2 increased *Cpt1a* gene expression [147,148].

As mitochondria plays vital roles in multiple cellular functions such as bioenergetics, biosynthesis, and apoptosis, dysfunctions in the mitochondrial quality control system are associated with the pathogenesis of several disease including metabolic diseases, cancers, and neurodegenerative disease [138,149]. Damaged mitochondria can be removed by mitophagy, a process consisting of selective sequestration of excessive or damaged mitochondria by the autophagosome and subsequent degradation by lysosome [150]. PTEN-induced kinase 1 (PINK1) and p62 are crucial players in mitophagy. Similarly to p62, the expression of PINK1 can be induced by oxidative stress in NRF2-dependent manner. Moreover, the increased PINK1 reduces oxidative stress-induced cytotoxicity by removing damaged mitochondria [151]. These findings suggested a potential role of Nrf2 in further controlling mitochondrial integrity via regulating the expression of p62 and PINK1.

Recently, multiple lines of evidence revealed that mitochondria plays pivotal roles in regulating the homeostasis of stem cells particularly in the context of bones [152]. As bone remodeling persists throughout life, the lineage commitments and dynamic interactions of two important stem cell populations—hematopoietic stem cells (HSCs) and mesenchymal stem cells (MSCs)—must be tightly regulated and finely tuned. Mitochondrial compromise has been shown to be closely associated with the pathogenesis of stem cell dysfunction in bone aging and pathologies [152]. It is understood that the fates of stem cells are influenced by metabolic status-glycolysis and OXPHOS [153]. In the skeletal system, the OBs differentiation from MSCs is mainly dependent on this metabolic switch. MSCs/osteogenic progenitors mostly rely on glycolysis, whereas mature OBs acquired energy by OXPHOS [154]. Significant upregulation of oxygen consumption rate and intracellular ATP level during osteoblastogenesis have been evidenced, indicating the involvement of mitochondrial energy metabolic switch in lineage commitment of MSCs. On the other hand, compromising of mitochondrial OXPHOS in MSCs by treatment with electron transport chain (ETC) inhibitor (e.g., antimycin A), uncoupler (e.g., FCCP), or ATP synthase inhibitor (e.g., oligomycin) leading to the suppression of osteoblastogenesis [155,156]. It is worthy of note that metabolic switch to OXPHOS could result in elevated presence of reactive oxidative species (ROS) as a byproduct, while concomitantly, differentiated OBs activate an antioxidant defense system, in particular SOD2, to prevent endogenous ROS accumulation [155]. SOD2 deficiency causes oxidative damage, impairs osteogenic differentiation and leads to the development of osteoporosis [156,157]. In addition, morphological alterations of mitochondria such as enlargement, elongation, and activation-related network formation are prominent features during osteoblastogenesis. As aforementioned, activation of TFAM, a transactivation target of NRF2, is crucial for mitochondrial functions, indicating the involvement of NRF2 in regulating mitochondria function and osteoblastogenesis. Moreover, activation of NRF2 activity with natural products ameliorate osteoporosis in rats [158,159]. These findings further support the notion that NRF2, the master regulator of antioxidant defense system, is crucial and positively associates with osteoblastogenesis.

HSCs, another major stem cell population in bone, participates in bone remodeling by differentiating into OCs. Similar to osteoblastogenesis, OC differentiation is accompanied by remarkable mitochondria increase and upregulation of mitochondrial metabolism [160]. Impairment of mitochondrial respiration by knocking out of mitochondrial complex I subunit Ndufs4 inhibits osteoclastogenesis and leads to osteopetrosis [161]. However, in contrast to osteoblastogenesis, knockdown of SOD2 both in vitro and in vivo increases mitochondrial ROS production and leads to accelerated osteoclastogenesis and reduction of bone volume [162]. Accumulating evidence supports that ROS are essential regulators of OCs lineage commitment from HSCs [102,163]. In the light of these findings, NRF2 clearly regulates mitochondrial function and ROS production in a sophisticated manner during osteoclastogenesis. Furthermore, NRF2 might also promote HSC lineage allocation toward OCs through regulating mitochondria biogenesis and mitochondrial quality, while restrain OCs from hyper-activation by fine-tuning ROS production.

## 11. The Role of NRF2 in Mitochondrial Regulation in Myeloma

As discussed in our previous report, NRF2 plays opposite roles in normal and cancer cells. Upregulation of NRF2 target genes were observed in chemoresistant and relapsed/refractory MM patients [108]. NRF2 activation has also been reported to contribute to proteasome inhibitors resistance in MM [15]. Increased NRF2 activity, which activates pro-survival signaling pathways, was observed in primary MM and MM cell lines treated with proteasome inhibitors. In line with the findings, genetic and pharmacologic inhibition of NRF2 reestablished the sensitivity of MM cells to bortezomib and carfilzomib [108,164]. In addition to drug resistance, NRF2 could affect MM cell growth and survival, as NRF2 inhibitors suppress MM cell growth and reduce the viability of MM cells [165,166].

Notably, elevated protein expression of NRF2 in carfilzomib-resistant MM cells was accompanied by increased level/expression of p62 and FAO [108]. These findings indicated that NRF2 could enhance mitochondrial function and quality in MM cells. Moreover, the expression of SOD2, mitochondria membrane potential, oxygen consumption rate, and ATP production were higher in bortezomib-resistant MM cells [167]. Recently, Zhan et al. demonstrated that gene expression of mitochondrial biogenesis signatures were significantly higher in relapsed and drug resistant myeloma samples than newly diagnosed patient samples. Inhibition of mitochondria OXPHOS dramatically reduced the growth of MM xenograft in in vivo model [168]. Similar results were reported by Alejandra Ortiz-Ruiz and colleagues [169]. These findings further demonstrated a pivotal role of mitochondria in drug-resistance of MM cells. Together, we reasoned that NRF2 could be involved with drug resistance through enhancing anti-oxidant and detoxification responses, and promoting mitochondria functions.

## 12. Concluding Remarks and Future Perspectives

Osteolytic bone lesions are one of the central features of MM, which leads to morbidity of patients. MBDs are caused by many factors from myeloma cells and the microenvironment that stimulate OCs to resorb bone and inhibit OB activity to increase bone mass. The dysfunction of the OC/OB axis in myeloma patients results in osteolytic lesions and/or osteoporosis, leading to bone pain, fracture, diminished bone survival, and reduced quality of life in patients. Many signaling pathways and factors were noted to be associated with MBDs. The role of the RANKL/OPG pathway was found to participate in MBD, and the identification of the RANKL/OPG pathway also led to the development of RANKL inhibitors for myeloma bone lesion therapy.

**Figure 4:**
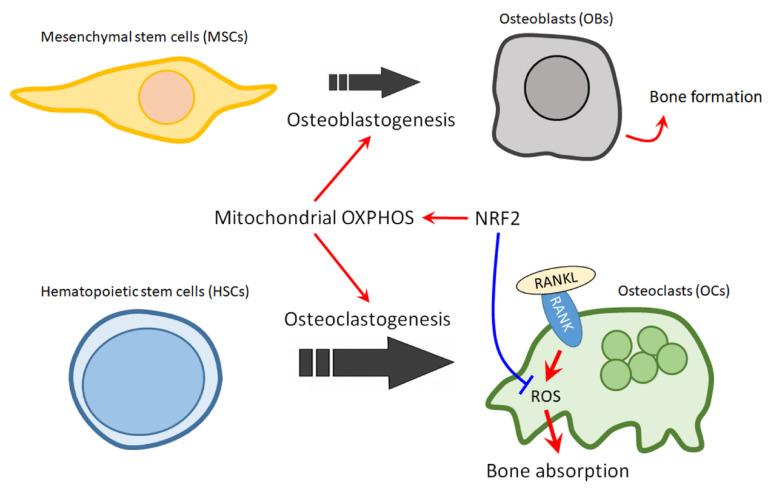
The role of NRF2 in mitochondrial regulation of bone homeostasis in MBD. The balance of OCs and OBs is impaired in osteolytic bone lesions, where OB activity is suppressed while the osteoclastogenesis is strongly upregulated. Activation of NRF2 could promote osteoblastogenesis, while restraining uncontrolled osteoclastogenesis. Red arrows indicate active effect; Blue bar arrow indicates suppressive effect.

NRF2, a master regulator of cellular defense system, has been considered as a target for chemoprevention and for cancer therapy. Here, we proposed that NRF2 can also be a therapeutic target for treating MBD. As mentioned, the balance of OCs and OBs is impaired in osteolytic bone lesions, where OB activity is suppressed while the osteoclastogenesis is strongly upregulated. Activation of NRF2 holds the potential for reversing the unbalance of OCs and OBs, since NRF2 could promotes osteoblastogenesis, while restrain uncontrolled osteoclastogenesis (Figure 4). Thus NRF2 activators should prove to be beneficial for MM patients with osteolytic lesions. However, as we have discussed above, NRF2 activation could have the opposite effect in BM environment cells and in MM cells. Thus, the effect of NRF2 activators on the growth and drug resistance of MM cells should be carefully noted. Nonetheless, the fact that cancer cells can activate NRF2 through several mechanisms including somatic mutations in Keap1 or NRF2 gene loci, hypermethylation at the promoter region of Keap1, transcriptional upregulation of the NRF2 gene through oncogene-dependent signaling, interruption of Keap1-NRF2 interaction, and the modification of Keap1 protein by electrophilic oncometabolites, may provide a possible resolution for this contradiction, in which cancer cells could lose their sensitivity to NRF2 activators [170,171]. In line with this notion, we have found that cells which expressed higher basal NRF2 protein level also have a weaker response to NRF2 activators. Therefore, it is worthy of further investigation for the effects of NRF2 activators on MBD in models with proteasome inhibitor-resistant MM cells or NRF2 hyperactivated MM cells. Owing to the elusive and complex nature of MM, more research is warranted to further investigate the specific role of NRF2 in myeloma bone lesions and to identify novel agents for NRF2 therapy. Nevertheless, for future clinical practice, we would propose that a test to profile the status of NRF2 signaling and/or the mutation in NRF2 signaling genes in MM cells collected from patient could be helpful to identify eligible population for NRF2 activator therapy for MBD. Furthermore, it is also possible that different classes of NRF2 activator should be used to treat MBD that were induced by MM cells with different NRF2 activation mechanisms.

## Abbreviations

ARE	antioxidant response elements
bZIP	basic leucine zipper protein family
β-TrCP	β-transducin repeat-containing protein
BMSCs	bone marrow stromal cells
CCL3	chemokine C-C motif 3
CBP	CREB-binding protein
CUL1-RBX1	Cullin 1-RING Box 1
CUL3-RBX1	Cullin 3-RING Box 1
DDK-1	dickkopf-1
DLX5	Distal-Less Homeobox 5
GCLC	glutamate-cysteine ligase catalytic
GSH	glutathione
GSTs	glutathione S-transferases
HSCs	Hematopoietic stem cells
IL-1,6	Interleukin-1,6
MIP-1-alpha	macrophage inflammatory protein 1
M-GCSF	macrophage-colony stimulating factor
MAPK	mitogen-activated protein kinase
MM	Multiple myeloma
Maf	Musculoaponeurotic fibrosarcoma
MDSCs	myeloid-derived suppressor cells
MBD	Myeloma bone disease
MSCs	Mesenchymal stem cells
NQO	NADPH-quinone oxidoreductase
RANKL	NF-kappa B ligand
NADPH	nicotinamide adenine dinucleotide phosphate
NFATc1	nuclear-factor-activated T-cell cytoplasmic 1
NRF2	Nuclear factor erythroid 2-related factor 2
OC-STAMP	osteoclast stimulatory transmembrance protein
OB	Osteoblast
OC	Osteoclast
OPG	Osteoprotegerin
OXPHOS	oxidative phosphorylation
PTHrP	parathyroid hormone-related protein
PTH	Parathyroid hormone
NQO1	NAD(P)H: quinone osidoreductase 1
RANKL	RANK ligand
ROS	reactive oxygen species
RXRα	retinoid X receptor alpha
RUNX2	Runt-related transcription factor 2
TRAP	tartrate-resistant acid phosphatase
TGF-beta	transforming growth factor beta
TNF-alpha	tumor necrosis factor-alpha
VCAM-1	vascular cell adhesion molecule-1
VEGF	vascular endothelial growth factor
